# Targeting ERBB2 mutations in solid tumors: biological and clinical implications

**DOI:** 10.1186/s13045-018-0630-4

**Published:** 2018-06-25

**Authors:** Sophie Cousin, Emmanuel Khalifa, Amandine Crombe, Yech’an Laizet, Carlo Lucchesi, Maud Toulmonde, Sylvestre Le Moulec, Céline Auzanneau, Isabelle Soubeyran, Antoine Italiano

**Affiliations:** 1 Early Phase Trials Unit, Institut Bergonié, 229 Cours de l’Argonne, 33000 Bordeaux, France; 2 Department of Medicine, Institut Bergonié, Bordeaux, France; 3 Department of Biopathology, Institut Bergonié, Bordeaux, France; 4 Department of Radiology, Institut Bergonié, Bordeaux, France; 5 Department of Bioinformatics, Institue Bergonié, Bordeaux, France

**Keywords:** ERBB2, Mutation, Targeted therapy, Secondary resistance

## Abstract

Preclinical data have shown that ERBB2 activating mutations are responsive to HER2 tyrosine kinase inhibitors. The aim of this study is to characterize the landscape of ERBB2 mutations in solid tumors and the potential efficacy of ERBB2 targeting.

We analyzed the next-generation sequencing results from 17,878 patients with solid tumors and reported the outcome of 4 patients with advanced ERBB2-mutated tumors treated with a combination of trastuzumab and lapatinib.

ERBB2 mutations occurred in 510 patients (2.85%). The tumor types with the highest incidence of ERBB2 mutations were the following: bladder (16.6%), small bowel (8.6%), ampullar (6.5%), skin non-melanoma (6.1%), and cervical cancer (5.5%). 49.4% (*n* = 282) were known as activating mutations. ERBB2 mutation was not mutually exclusive of ERBB2 amplification which occurred in up to 10% of cases. PI3KCA activating mutations were associated with ERBB2 mutations in 12.4% of cases mainly in breast and lung cancer. Four patients (endometrial, colorectal, cholangiocarcinoma, and adenosarcoma of the uterus) were treated with a combination of trastuzumab and lapatinib. All of them experienced tumor shrinkage resulting in stable disease in three cases and partial response in one case. One patient developed secondary resistance. Sequencing of the progressing metastasis allowed the identification of the ERBB2 L869R mutation previously associated with resistance to lapatinib in vitro.

These results support further clinical investigation aiming to demonstrate that *ERBB2*-mutational driven therapy can improve patient care irrespective of histology.

**Electronic supplementary material:**

The online version of this article (10.1186/s13045-018-0630-4) contains supplementary material, which is available to authorized users.

To the Editor

We investigated the incidence of *ERBB2* mutations in a large panel of tumors analyzed by next-generation sequencing (NGS) and reported the clinical impact of *ERBB2* targeting in this setting as well as potential mechanisms of secondary resistance.

We analyzed the AACR Project Genomics Evidence Neoplasia Information Exchange (GENIE) [1] and the Bergonie Institute Profiling study (ClinicalTrials.gov Identifier: NCT02534649) [2] databases (see Additional file 1: Supplementary Methods).

Seventeen thousand eight hundred seventy-eight patients were included in the study. Figure 1a describes the distribution of tumor types. Five hundred seventy-one *ERBB2* mutations involving all the domains of the receptor (Fig. 1b) were found in 510 patients (2.85%): 472 missense mutations, 66 in frame mutations, 14 fusions, 11 frame shift mutations, 5 non-sense, and 3 splice mutations. Figure 1c describes the incidence of mutations according to tumor types. 49.4% (*n* = 282) of the mutations identified were described as oncogenic according to COSMIC and were more frequently detected in the bladder (9.4%), small bowel (7.1%), ampullar (6.5%), cervical cancer (4.1%), and nerve sheath tumor (2.9%), respectively (Fig. 1d). The most common mutations are represented in Table 1.

**Fig. 1:**
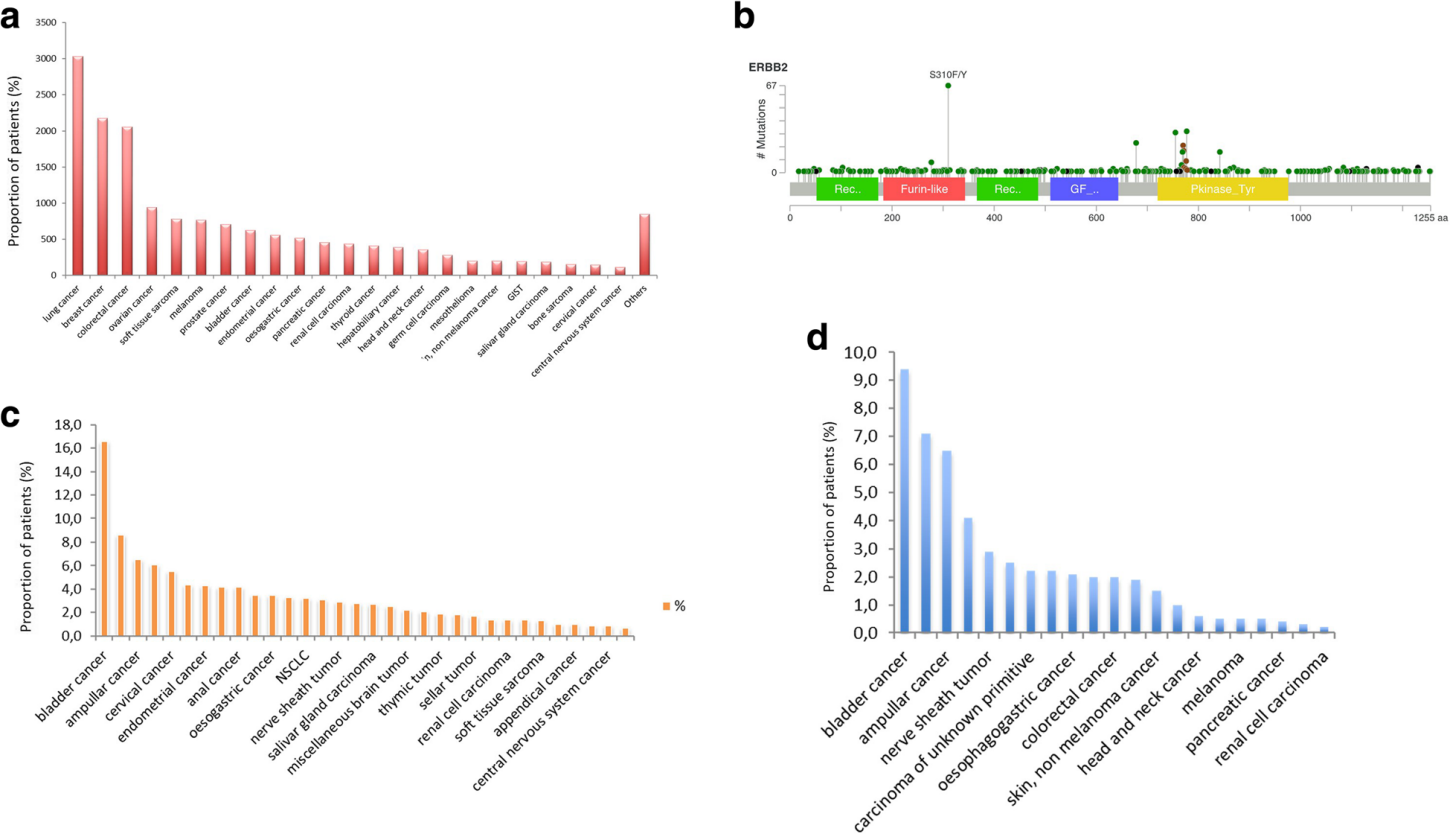
Landscape of ERBB2 mutations in cancer. **a** Proportion of histological subtypes included in the study. **b** ERBB2 hotspot mutations. **c** Proportion of patients with ERBB2 mutations according to the histological subtype. **d** Proportion of patients with activating ERBB2 mutations according to the histological subtype

**Table 1:** Most common ERBB2 mutations in cancer patients

Mutation	Frequency in GENIE database (%)	Proportion of ERBB2 mutations (%)	Type of mutation	Function
S310F/Y	0.46	12.6	Missense	Activating
Y772_A775dup	0.21	6.9	In Frame	Activating
R678Q	0.17	4.5	Missense	Activating
L755S/A/P	0.17	5.5	Missense	Activating
V777L/M	0.12	4.0	Missense	Activating
V842I	0.09	3.1	Missense	Activating
D769Y/H/N	0.09	2.9	Missense	Inactivating

The three most frequent alterations co-occurring with *ERBB2* mutations were the following: *TP53* mutation (*n* = 557, 59.5%), *CDK12* amplification (*n* = 216, 24%), and *PI3KCA* mutation (*n* = 200, 21.4%).

Among the 39 tumors bearing both *ERBB2* amplification and mutation, 16 (32%) were breast ductal carcinoma, 8 (16%) lung adenocarcinoma, and 6 (12%) bladder urothelial carcinoma.

Four patients with *ERBB2* mutation-bearing tumor were treated with dual *ERBB2* blockade with trastuzumab + lapatinib and experienced clinical benefit (Additional file 1: Table S2 and Figure S1). Despite tumor shrinkage at first tumor assessment (− 27%), patient 4 presented with secondary resistance with the occurrence of unique brain metastasis requiring neurosurgery. The molecular analysis of the resected brain metastasis revealed the presence of a L869R ERBB2 mutation, recently described as a mutation of resistance to lapatinib, whereas the two activating *ERBB2* mutations present in the primary tumor were not identified [3].

The recent results from the SUMMIT study have shown that clinical benefit from ERBB2 tyrosine kinase inhibitor (neratinib) may be dependent on the type of ERBB2 alteration and type of tumor. For instance, no clinical activity was observed in bladder and colorectal cancers [4]. Of note, the patient with colorectal cancer included in our study had significant tumor shrinkage with a combination of anti-HER monoclonal antibody and of tyrosine kinase inhibitor. Further studies are needed to investigate if genomic determinants of response may differ when a monoclonal antibody is added to a tyrosine kinase inhibitor in ERBB2-mutated patients.

Interestingly, *PI3KCA* mutations represent one of the most frequent co-alterations identified in *ERBB2*-mutated tumors. The PI3K/Akt/mTOR pathway has been shown to play a potential important role in resistance to *ERBB2* targeting therapy in *ERBB2*-overexpressing breast cancer. Moreover, preclinical studies indicated that inhibitors of this pathway can act synergistically with trastuzumab in resistant models [5]. This finding should be taken in consideration in ongoing and future trials investigating *ERBB2*-targeted therapy in *ERBB2*-mutated tumors.

Unfortunately, targeted therapies suffer from a major limitation, that is, the duration of any observed clinical benefit is invariably limited in length, owing to the relatively rapid acquisition of drug resistance. We report here for the first time a case of a ERBB2 activation loop mutation in a patient with non-amplified ERBB2 mutant colorectal cancer with acquired resistance to trastuzumab combined with lapatinib. The L869R mutation is located within the activation loop of the kinase domain and associated with gain of function activity. This mutation, which was identified in the secondary progressing brain lesion was not present in the primary tumor, was shown to confer resistance to lapatinib in vitro but to be sensitive to second-generation *ERBB2*/EGFR inhibitor such as neratinib in the clinical setting [6]. Of note, we cannot exclude the possibility that the L869R mutant cells might be already present in a minority of clones of the primary tumor (given tumor heterogeneity and limits in the sensitivity of NGS technologies) and selected under pressure of trastuzumab and lapatinib combination treatment.

The significant mutation rate of *ERBB2* in several tumor types and the promising preliminary activity of dual *ERBB2* targeting reported here deserved further clinical investigation aiming to demonstrate that *ERBB2*-mutational driven therapy can improve patient care irrespective of histology.

## Additional file

Additional file 1: Supplementary Methods and Results. (DOCX 622 kb)

## References

[1] Rose S (2016). Huge data-sharing project launched. Cancer Discov.

[2] Cousin S, Grellety T, Toulmonde M, Auzanneau C, Khalifa E, Laizet Y (2017). Clinical impact of extensive molecular profiling in advanced cancer patients. J Hematol Oncol.

[3] Biswas R, Gao S, Cultraro CM (2016). Genomic profiling of multiple sequentially acquired tumor metastatic sites from an “exceptional responder” lung adenocarcinoma patient reveals extensive genomic heterogeneity and novel somatic variants driving treatment response. Cold Spring Harb Mol Case Stud.

[4] Hyman DM, Piha-Paul SA, Won H, Rodon J, Saura C, Shapiro GI, Juric D, Quinn DI, Moreno V, Doger B, Mayer IA, Boni V, Calvo E, Loi S, Lockhart AC, Erinjeri JP, Scaltriti M, Ulaner GA, Patel J, Tang J, Beer H, Selcuklu SD, Hanrahan AJ, Bouvier N, Melcer M, Murali R, Schram AM, Smyth LM, Jhaveri K, Li BT, Drilon A, Harding JJ, Iyer G, Taylor BS, Berger MF, Cutler RE, Xu F, Butturini A, Eli LD, Mann G, Farrell C, Lalani AS, Bryce RP, Arteaga CL, Meric-Bernstam F, Baselga J, Solit DB (2018). HER kinase inhibition in patients with HER2- and HER3-mutant cancers. Nature.

[5] Lopez S, Cocco E, Black J (2015). Dual ERBB2/PIK3CA targeting overcomes single-agent acquired resistance in ERBB2-amplified uterine serous carcinoma cell lines in vitro and in vivo. Mol Cancer Ther.

[6] Hanker AB, Brewer MR, Sheehan JH, Koch JP, Sliwoski GR, Nagy R (2017). An acquired *ERBB2*T798I gatekeeper mutation induces resistance to neratinib in a patient with *ERBB2* mutant-driven breast cancer. Cancer Discov..

