# Supplementary material for: Targeting ERBB2 mutations in solid tumors: biological and clinical implications
Source: J Hematol Oncol. 2018 Jun 25;11:86. doi: 10.1186/s13045-018-0630-4 (PMC6019715; doi:10.1186/s13045-018-0630-4)
Supplement: Supplementary file 1 — Supplementary Methods and Results. (DOCX 622 kb) [file 13045_2018_630_MOESM1_ESM.docx]

**Supplementary Methods and Results**

**Supplementary Methods**

The analysis was based on sample and mutation annotation tables available in GENIE via Synapse data portal (synapseid:syn7222066, at <https://www.synapse.org/#!Synapse:syn7222066/files/>). Patients with tumors bearing potential targetable ERBB2 mutations were identified and then patients with hematological malignant disease were excluded.

Additionally, we reported cases of four patients included in the Bergonie Institute Profiling study (ClinicalTrials.gov Identifier: NCT02534649), as previously described [1]. These patient cases were discussed during a weekly molecular multidisciplinary board. Because they were out of standard treatment possibility for their metastatic disease, the decision was made to propose them a compassional treatment with a dual ERBB2 blockade with Trastuzumab + Lapatinib. Trastuzumab was given intraveinously with a charging dose of 8 mg/kg and then 6 mg/kg every 3 weeks. Lapatinib was given per os at 1250mg daily, continously. Response to treatment was evaluated using RECIST 1.1, and using when available FGD-PET results. This study was approved by the Institutional Review Board.

Supplementary references

1. Cousin S, Grellety T, Toulmonde M, Auzanneau C, Khalifa E, Laizet Y, et al. Clinical impact of extensive molecular profiling in advanced cancer patients. J Hematol Oncol. 2017; 10:45.

| **Table S1. Patients characteristics** | | | | | | | | |
| --- | --- | --- | --- | --- | --- | --- | --- | --- |
| **Patient** | **Gender** | **Age** | **Cancer** | **Location of the biopsy for molecular screening** | **HER2 mutation** | **Number of previous lines** | **TTP under treatment**  **(months)** | **Best response according to RECIST** |
| 1 | F | 62 | Endometrial carcinoma with peritoneal and lymph nodes locations | Primary tumor | Exon 21 :V842I | 4 | 8.9 | SD (+3%) |
| 2 | F | 73 | Cholangiocarcinoma with hepatic, lymph nodes and pulmonary locations | Primary tumor | Exon 8 : Ser310TYR | 4 | 7.1 | SD (-10.5%) |
| 3 | F | 66 | Uterine adenosarcoma with bone, pulmonary and brain locations | Primary tumor | Exon 20 :V777L | 2 | 8.2 | PR (-41%) |
| 4 | M | 48 | Colorectal adenocarcinoma with hepactic, pulmonary, peritoneal and lymph nodes location | Primary tumor  Brain metastasis | Exon 20 : V777L  Exon 21 : V842I  Exon 21 : L869R | 2 | 3.5 | SD (-27%) |

**
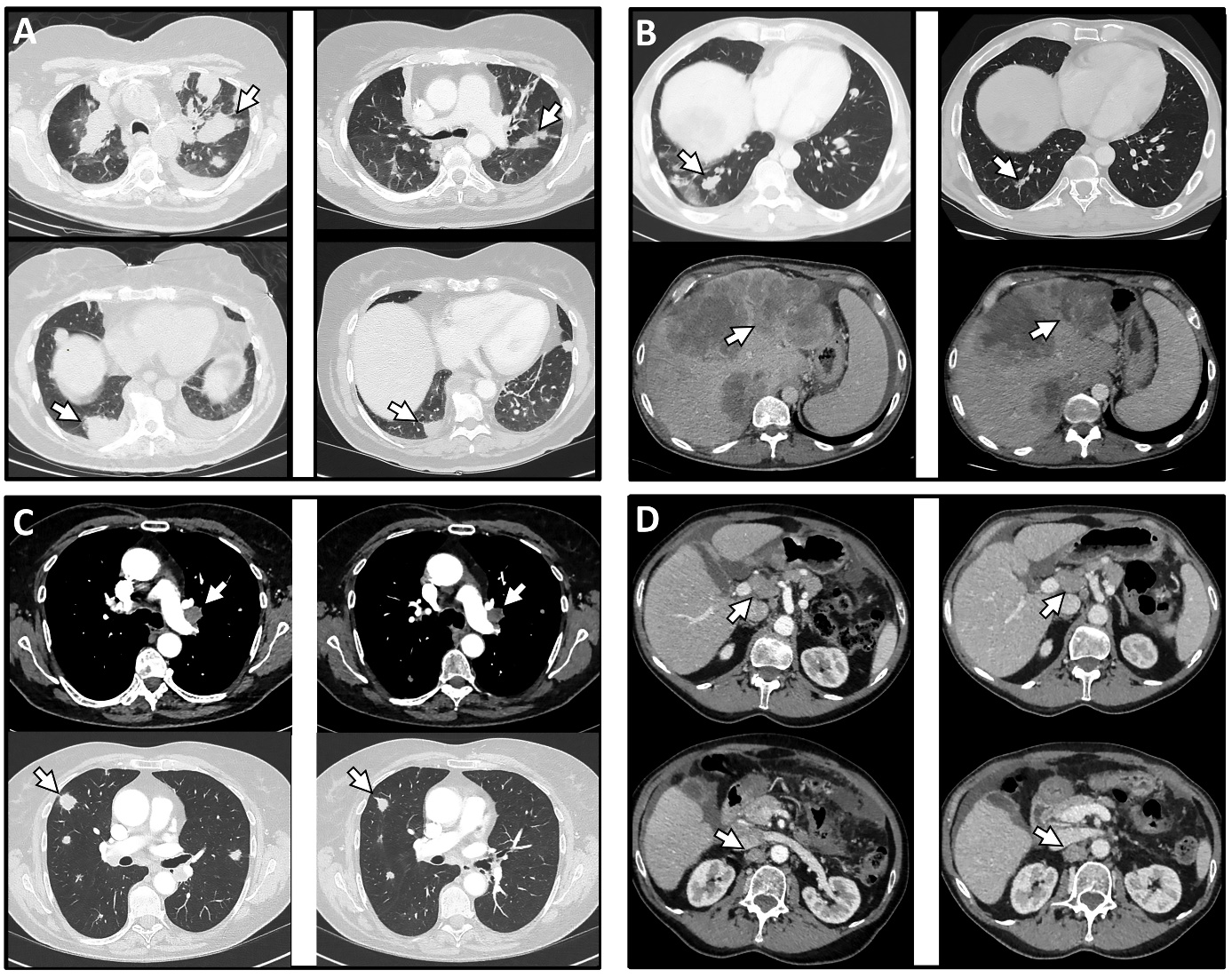
**

**Figure S1**. Anti-tumor activity of dual ERBB2 blockade with trastuzumab and lapatinib in ERBB2 mutated cancer patients. A-B: Shrinkage of lung metastases at week 6 (B) of treatment in patient with advanced colorectal cancer (A) (patient 4) C-D: Partial response observed at week 8 of treatment (D) in patient with advanced uterine adenosarcoma (C) (patient 3)
